# Supplementary material for: Research on the promoting effect of servitization on export technological sophistication of manufacturing enterprises
Source: PLoS One. 2021 Aug 31;16(8):e0255891. doi: 10.1371/journal.pone.0255891 (PMC8407592; doi:10.1371/journal.pone.0255891)
Supplement: Attachment — Submitted filename: Reviewer Comment_PONE-D-20-40809.docx [file pone.0255891.s001.docx]

**Introduction**
Research on the Promoting Effect of Servitization on Export Technological Sophistication of Manufacturing Enterprises; starting from the introduction, the section is well written. In the current version, the paragraph is strong: it does provide enough information about the research topic, the reference context, and the gap that the work wants to fill. The function of the introduction is to describe the reference context from which the research question originates and to highlight the research gap to give evidence of the contribution of the paper. In the actual version of the paragraph, this logical sequence is well introduced and adequately supported. So, the entire section is well written, highlighting the relevance of the theme, the gap in literature and the establishment of consequent research aims.

**Theoretical framework and literature review**
In general terms, the paper has fundamental theoretical framework and literature review, started with background and hypothesis building. And indeed, there is evidence of organization and clarity.

**Hypotheses**
In general, all proposed hypothesis is adequately supported. All the hypotheses are supported by the references linking it to the author's work objectives. The moderating effect of GVC participation s well described and adequate.

**Methodology and Design**
The explanation of measurement variables is done in good length and precise. Adequate data analyses to arrive at the results indicating robustness of the analyses.

**Conclusion**
The paragraph conclusion is adequate in the current form.

In the paper, there is no section dedicated to the theoretical and practical implications. More details are needed about (a) research limitations, and (b) the managerial and theoretical implications. Theoretical and managerial implications could be more extensively debated consistent with the results obtained.
