# Supplementary material for: Research on the promoting effect of servitization on export technological sophistication of manufacturing enterprises
Source: PLoS One. 2021 Aug 31;16(8):e0255891. doi: 10.1371/journal.pone.0255891 (PMC8407592; doi:10.1371/journal.pone.0255891)
Supplement: Attachment — Submitted filename: Response to Reviewers.docx [file pone.0255891.s002.docx]

**Response to Reviewers**

Response to Reviewer #1:

Thank you for your helpful suggestions on this article

In the Conclusions and Discussions part, we have adopted your suggestion and added the content of the study limitations. The theoretical and practical implications of the study were mentioned in the original paper, and before resubmission, we further clarified them and improved the coherence with the obtained conclusions.

Response to Reviewer #2:

Thank you for your helpful suggestions on this article.

Response to - 3. Research design

We provide detailed definitions and explanations of econometric concepts to help readers understand the disciplinary basis of our modeling, "Econometrics is a discipline that uses mathematical and statistical methods to determine specific quantitative relationships in economic relationships. Econometrics measures and validates actual statistics of economic relationships and provides quantitative information for the qualitative account of economic theory on the dependence of economic variables to predict the future and provide a scientific basis for economic planning and determination of economic policy. In the subsequent study, we construct models based on econometric principles."

Response to -- 3.1 Econometrical model specification

Due to an oversight, we did not have an explanation of these parameters in the original draft, but we have now added an explanation of this section, "For estimated parameters, $\alpha_{0}$ denotes the intercept term, or constant term is used to fit the model and has no practical significance. $\beta_{1}$ and $\beta_{2}$ denote the estimated parameters of the primary and secondary terms of the core variable manufacturing servitization, respectively. $\gamma$ denotes estimated parameters representing the set of control variables. For the estimated parameters of the fixed effects, $\kappa_{i}$, $\mu_{p}$and $\eta_{t}$ denote the industry level, provincial level, and year level fixed effects and random errors, respectively. $\varepsilon_{fit}$ denotes the random perturbation terms."

Besides, we add specific literature to clarify the reference base for control variables.

Response to - 4. Empirical results and analysis

At the request of the reviewers, we added Figures in several parts of the article to facilitate the reader's understanding of the study through visualization. In the Indicator measure part, the Mediation effect analysis part, the Heterogeneity results and analysis part, and the Expansibility analysis part, a figure was added respectively.
