# Supplementary material for: Research on the promoting effect of servitization on export technological sophistication of manufacturing enterprises
Source: PLoS One. 2021 Aug 31;16(8):e0255891. doi: 10.1371/journal.pone.0255891 (PMC8407592; doi:10.1371/journal.pone.0255891)
Supplement: Attachment — Submitted filename: Response to Reviewers.docx [file pone.0255891.s003.docx]

Response to the Academic Editor

Dear editor,

We checked the reference list and confirmed its correctness and completeness. In addition, we added two additional publications due to the reviewer's suggestion and marked them in red in the list.

Response to Reviewers

Dear Reviewer #3,

Thank you for your constructive suggestions for the improvement of the manuscript. According to your suggestions and some of your questions, we reply to them one by one.

Q1: The full name of the abbreviations should be given when it is used at the first time, such as GVC, HS.

R1: We show the first appearance of a term by its full name. (P1, P7)

Q2: "With the comparative advantage of low production costs, China has gained the rapid growth of exports." Can you provide a reference for this sentence?

R2: We provide a literature basis for this sentence. (P2)

Q3: Can you provide a more detailed illustration for the computation of HHIit, and provide short words to show the meaning of DTSIfit.

R3: We provide a more detailed explication of the dependent variable DTSI for the model. $DTSI_{fit}$denotes export technological sophistication of enterprises, as one of the important indicators of export performance, it measures the level of export product quality of enterprises in a narrow sense and the export competitiveness of enterprises in a broad sense (P5). Besides, we provide a more detailed illustration for the computation of HHI. A Herfindal index formula $HHI_{it}=\sum_{s\in Ind_{j}} (SALE_{ist}/SALE_{st})^{2}=\sum_{s\in Ind_{j}} MS_{ist}^{2}$. In this formula, the sum of the squares of the share of sales of each industry in the total sales of a region constitutes the degree of industrial agglomeration in that region. When the proportion of a certain industry is larger, the final $HHI_{it}$value will also be larger. (P6).

Q4: In the last paragraph of section “Econometrical model specification”, “For the estimated parameters of the fixed effects……”， the number of subject and object are not consistent.

R4: The original sentence was incorrectly translated before, and we correct it. We thank the reviewers for pointing out this error. (P6)

Q5: Can you supplement the information on the software and tools which was used to construct your models (mediation effect analysis, Heckman two-step method) and in your statistical works in your manuscript? Such as version. More information (code) can be freely accessed?

R5: Throughout the manuscript, we use the statistical software StataMP16.1 to measure $DTSI_{it}$, $SR_{it}$ and other control variables. In addition, we use this software to perform panel fixed effects regressions on the models constructed in the whole manuscript, including mediating effects regressions to discriminate the impact mechanism and Heckman-Two Step method regressions to discriminate endogeneity (P9). The availability of the code is not publicly available because it is still a part of our phase study, but we provide detailed information on the steps for the metric measurement and model regression in the manuscript.

Q6: "Observation" in the header of Table 1 means the number of entries? Then the procedures used for missing variables can be shown? Ref. 35 has been downloaded, but I did not find out which steps were adopted by them to preprocess missing indicators.

R6: "Observation" that appears in each table indicates the entry used for the study. Because the presence of missing values for each entry is not allowed in the panel regression, the statistical software Stata automatically eliminates entries with missing values from the model during the regression process, so the number of entries presented in **Table 1** does not necessarily equal the number of entries in the subsequent tables of the regression results (P9). Besides, the original reference was cited incorrectly and we have corrected it, for which we are grateful. In fact, we fully refer to Brandt et al. (2012) and Feenstra et al. (2014). However, it should be noted that in modeling regressions, the statistical software automatically eliminates a small number of entries that may lead to covariance due to the introduction of multidimensional fixed effects (P8).

Q7: Please provide the concrete definition or reference for "adjusted enterprise".

R7: In the manuscript, the term "adjusted enterprise" is intended to refer to export technological sophistication at the enterprise level after the product quality has been corrected, not to the enterprise itself. (P7)
